# Supplementary material for: Anoxygenic photosynthesis and the delayed oxygenation of Earth’s atmosphere
Source: Nat Commun. 2019 Jul 9;10:3026. doi: 10.1038/s41467-019-10872-z (PMC6616575; doi:10.1038/s41467-019-10872-z)
Supplement: Supplementary file 1 — Supplementary Information [file 41467_2019_10872_MOESM1_ESM.pdf]

Supplementary Information for

Anoxygenic photosynthesis and the delayed oxygenation of Earth's atmosphere

Kazumi Ozaki et al.

a. b.

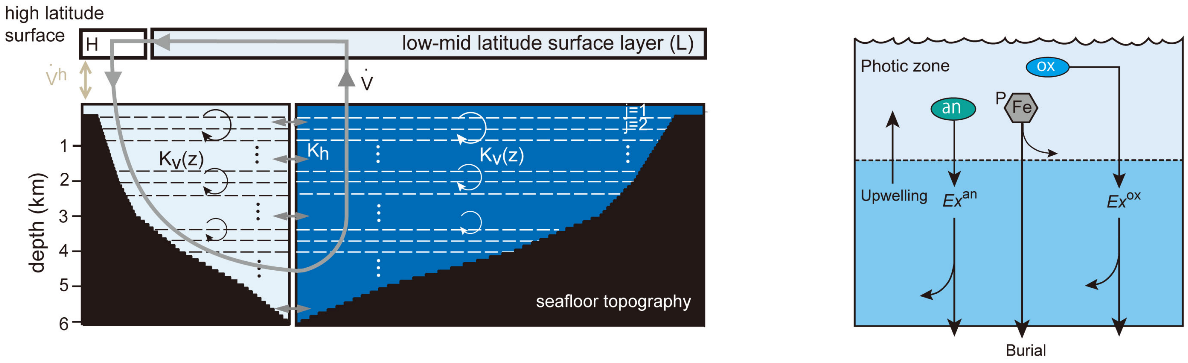

Supplementary Figure 1. Schematic figure of ocean configuration in the CANOPS-KB model (a). Ocean interior below the surface layers is divided into low-mid and high latitude sectors in the proportion of 0.75:0.25. Both sectors are vertically divided into 60 layers (j) to a maximum depth of 6100 m, with a layer spacing of 100m. Each sector interacts with modern ocean bathymetry. The model includes water transport by thermohaline circulation ($\dot{V}$) and vertical/horizontal eddy diffusion (K_v, K_h). Shown in (b) is a schematic depiction of the competitive photosynthesis scheme discussed in the text, where 'ox' represents oxygenic phototrophs, 'an' represent anoxygenic phototrophs, 'Fe' shows iron oxides (with associated scavenged P), and Ex^i terms represent carbon export fluxes by oxygenic (ox) and anoxygenic (an) phototrophs.

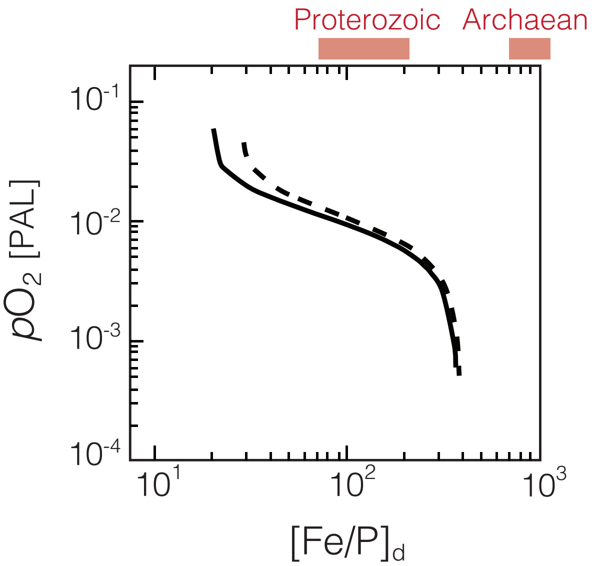

Supplementary Figure 2. Effect of varying phosphorus flux to the ocean on the stability analysis presented in the Main Text. Shown are equilibrium atmospheric pO_2 values as a function of deep ocean Fe/P ratio ($[Fe/P]_d$) for our reference case (solid) and a model ensemble in which we reduce phosphorus input by 50% (dashed). Note that in these simulations we also reduce global erosion rates (and thus organic carbon oxidation) by a similar factor (i.e., $f_w = 0.5$). Under the physically unrealistic assumption that these two factors are uncoupled, the equilibrium atmospheric pO_2 under a given set of boundary conditions would be revised slightly downward.

Supplementary Table 1. Genes involved in phosphorus metabolism in *C. phaeoferrooxidans* strain KB01.

Gene	Number of copies (ORF ID #)
PhoB (phosphate regulon response regulator)	2 (*22_16*), (*113_3*)
PhoR (phosphate regulon sensor)	1 (*22_15*)
PhoU (phosphate uptake regulator)	5 (*107_14*), (*62_3*), (*63_3*), (*63_4*), (*64_0*)
PhoH (phosphate starvation-inducible protein)	1 (*58_9*)
PstC (phosphate ABC transporter)	4 (*107_11*), (*62_0*), (*63_0*), (*64_3*)
PstA (phosphate ABC transporter)	4 (*107_12*), (*62_1*), (*63_1*), (*64_2*)
PstB (phosphate transporter ATP-binding protein)	4 (*107_13*), (*62_2*), (*63_2*), (*64_1*)
PstS (phosphate-binding protein)	6 (*113_1*), (*110_6*), (*107_10*), (*64_10*), (*90_4*), (*47_6*)
Alkaline phosphatase (PhoA)	2 (*113_6*), (*44_1*)
Polyphosphate kinase	2 (*113_5*), (*44_19*)

Supplementary Table 2. Parameter definitions and default parameter values for the 1-D water column model.

Parameter	Description	Default Value	Units	Source
**cyanobacteria**				
μ_c	maximum growth rate	0.004	µmolC kg^{-1} s^{-1}	[10]
I_c	half-saturation constant for light-limited growth	98	µmol photons m^{-2} s^{-1}	[10]
$K_{c,P}$	half-saturation constant for nutrients	0.015	µmol kg^{-1}	[10]
**photoferrotrophs**				
μ_p	maximum growth rate	0.002	µmolC kg^{-1} s^{-1}	This study
I_p	half-saturation constant for light-limited growth	1	µmol photons m^{-2} s^{-1}	[11]
$K_{p,Fe}$	half-saturation constant for dissolved Fe	11	µmol kg^{-1}	This study
$K_{mp,P}$	half-saturation constant for nutrients	0.005	µmol kg^{-1}	This study
**physical parameters**				
T	water temperature	25	ºC	-
I	ionic strength	0.7	-	-
I_0	incident light flux	1300	µmol photons m^{-2} s^{-1}	[10]
$1/\lambda$	light attenuation length scale	15	m	[12]
w	upwelling rate	0.5	m d^{-1}	-
K_v	eddy diffusivity	10^{-4}	m^2 s^{-1}	-
K_d^{FeP}	distribution coefficient for P on Fe oxides	0.025	µM^{-1}	[13]

Supplementary Table 3. Major reactions in the CANOPS-KB model. OM represents $(CH_2O)_\alpha(NH_4^+)_\beta H_3PO_4$.

Process	Stoichiometry
Ammonia assimilation	$\alpha CO_2 + \beta NH_4^+ + H_3PO_4 + \alpha H_2O \rightarrow OM + \alpha O_2$
Nitrate assimilation	$\alpha CO_2 + \beta NO_3^- + H_3PO_4 + (\alpha + \beta)H_2O + 2\beta H^+ \rightarrow OM + (\alpha + 2\beta)O_2$
Aerobic respiration	$OM + \alpha O_2 \rightarrow \alpha CO_2 + \beta NH_4^+ + H_3PO_4 + \alpha H_2O$
Denitrification	$OM + \frac{4}{5}\alpha NO_3^- + \frac{4}{5}\alpha H^+ \rightarrow \alpha CO_2 + \beta NH_4^+ + H_3PO_4 + \frac{7}{5}\alpha H_2O + \frac{2}{5}\alpha N_2$
Sulphate reduction	$OM + \frac{1}{2}\alpha SO_4^{2-} + \alpha H^+ \rightarrow \alpha CO_2 + \beta NH_4^+ + H_3PO_4 + \alpha H_2O + \frac{1}{2}\alpha H_2S$
Nitrification	$NH_4^+ + 2O_2 \rightarrow NO_3^- + H_2O + 2H^+$
Aerobic H_2S oxidation	$\Sigma H_2S + 2O_2 \rightarrow SO_4^{2-} + 2H^+$

$\sum H_2S = H_2S + HS^-$

Supplementary Table 4. Biogeochemical formulations used in the CANOPS-KB model. l and h represent low- and high-latitude surface layer, respectively. z is water depth, and w is upwelling rate at $z = h_m$.

Parameter	Units	Formulation
Total export production	mol C m^{-2} yr^{-1}	$J_{ex}^{total} = \alpha \cdot h_m \cdot \varepsilon \cdot [PO_4^{3-}] \cdot \frac{[PO_4^{3-}]}{K_m^P + [PO_4^{3-}]}$
New production of photoferrotrophs	mol C m^{-2} yr^{-1}	$J_{ex}^{pfe} = \min\left\{J_{Fe}^{up} / r_{FeC}, J_{ex}^{total\,l}\right\}$
New production of cyanobacteria	mol C m^{-2} yr^{-1}	$J_{ex}^{cyano} = \begin{cases} J_{ex}^{total\,l} - J_{ex}^{pfe} & : \text{Fe-limit}\,(J_{ex}^{pfe} < J_{ex}^{total\,l}) \\ 0 & : \text{P-limit}\,(J_{ex}^{pfe} = J_{ex}^{total\,l}) \end{cases}$
Aerobic respiration	mol C m^{-3} yr^{-1}	$R_{O_2} = (\sum k_i G_i) \cdot \frac{[O_2]}{K_{O_2} + [O_2]}$
Denitrification	mol C m^{-3} yr^{-1}	$R_{deni} = (\sum k_i G_i) \cdot \frac{K'_{O_2}}{K'_{O_2} + [O_2]} \cdot \frac{[NO_3^-]}{K_{NO_3} + [NO_3^-]}$
Sulphate reduction	mol C m^{-3} yr^{-1}	$R_{MSR} = (\sum k_i G_i) \cdot \frac{K'_{O_2}}{K'_{O_2} + [O_2]} \cdot \frac{K'_{NO_3}}{K'_{NO_3} + [NO_3^-]} \cdot \frac{[SO_4^{2-}]}{K_{SO_4} + [SO_4^{2-}]}$
Nitrification	mM yr^{-1}	$R_{nitrf} = k_{NH_4} \cdot [NH_4^+] \cdot [O_2]$
Sulphide oxidation	mM yr^{-1}	$R_{H2Sox} = k_{H_2S} \cdot [\Sigma H_2S] \cdot [O_2]$
Fe(II) upwelling flux	mol Fe m^{-2} yr^{-1}	$J_{Fe}^{up} = [Fe/P]_d \cdot J_P^{up}$
P scavenging and burial	mol P m^{-2} yr^{-1}	$J_{scav} = \begin{cases} \gamma \cdot K_d^{FeP} \cdot [PO_4^{3-}]_l \cdot J_{Fe}^{up} & \text{when}\,[O_2]_{j=1} < 1\,\mu M \\ 0 & \text{when}\,[O_2]_{j=1} \geq 1\,\mu M \end{cases}$
Phosphate upward flux	mol P m^{-2} yr^{-1}	$J_P^{up} = A_{j=1} w \cdot [PO_4^{3-}]_{j=1} + A_{j=1} K_v \left.\frac{\partial [PO_4^{3-}]}{\partial z}\right\|_{z=h_m}$
C_{org} burial efficiency	%	$BE_{oc} = \frac{be_1 - be_2}{1 + SR/a} + be_2$
P_{org} burial efficiency	%	$BE_{Porg} = BE_{oc}^* \cdot (1 + \exp(-0.001/SR))^{-1} \cdot \left(\alpha_P + (1-\alpha_P)\frac{[O_2]_{bw}}{[O_2]_{bw}^*}\right)$
Fe-bound P burial	%	$BE_{Fe\text{-}bound} = BE_{oc}^* \cdot (1 + \exp(-0.001/SR))^{-1} \cdot \frac{[O_2]_{bw}}{[O_2]_{bw}^*}$
Authigenic P burial	%	$BE_{auth} = 2 \cdot BE_{oc}^* \cdot (1 + \exp(-0.001/SR))^{-1}$

Supplementary Table 5. Constants used in the CANOPS-KB model.

Parameter	Symbol	Units	Value
**physical parameters**			
Ocean surface area	A	m^2	3.62×10^{14}
Coastal area (z < -200 m)	A_{cs}	m^2	0.271×10^{14}
Surface area of high-latitude layer (H)	A_h	m^2	3.62×10^{13}
Depth of mixed layer	h_m	m	100
Grid spacing	Δz	m	100
Water depth of ocean bottom	z_b	m	6100
Ocean overturning rate	V	Sv	20
High-latitude convection	V_h	Sv	57.4
Horizontal diffusion coefficient	K_h	$m^2\ s^{-1}$	1000
**biogeochemical parameters**			
Efficiency factor for phosphate uptake at L	ε_l	y^{-1}	1.0
Efficiency factor for phosphate uptake at H	ε_h	y^{-1}	0.15
Phosphate half saturation constant	Km^P	mM	1×10^{-6}
Redfield ratio for C and P	α	mol mol^{-1}	106
Redfield ratio for N and P	β	mol mol^{-1}	16
Fe/C stoichiometry of photoferrotrophy	r_{FeC}	mol mol^{-1}	4
Distribution coefficient for phosphorus scavenging	K_d^{FeP}	mM^{-1}	70
Preservation efficiency of scavenged P	γ		0.5
POM sinking velocity	v_{POM}	m d^{-1}	100
Weight fraction of G_1	f_{G1}		0.72
Weight fraction of G_2	f_{G2}		0.25
Weight fraction of G_3	f_{G3}		0.03
Decomposition rate of G_1	k_1	d^{-1}	0.6
Decomposition rate of G_2	k_2	d^{-1}	0.1
Decomposition rate of G_3	k_3	d^{-1}	0.0
Aerobic respiration of O_2 half saturation constant	K_{O2}	mM	8×10^{-3}
Denitrification half saturation constant	K_{NO3}	mM	3×10^{-2}
Half saturation constant for sulphate reduction	K_{SO4}	mM	0
Ammonium oxidation rate	k_{NH4}	$mM^{-1}\ y^{-1}$	1.825×10^4
Sulphide oxidation rate	k_{H2S}	$mM^{-1}\ y^{-1}$	3.65×10^3
Riverine reactive phosphorus input rate	R_P	Tmol P y^{-1}	0.18
Riverine nitrogen input rate	R_N	Tg N y^{-1}	0
Atmospheric nitrogen deposition	A_N	Tg N^{-1}	0
Baseline C_{org} weathering flux	$J_{w,0}^{org}$	Tmol C y^{-1}	10.5
C_{org} burial limit at zero sedimentation	be_1	%	0.5 for $[O_2]_{bw}$ > 200 μM<br>50 for $[O_2]_{bw}$ < 30 μM
C_{org} burial limit at infinite sedimentation	be_2	%	75
Center of regression for C_{org} burial efficiency	a	cm y^{-1}	0.264 for $[O_2]_{bw}$ > 200 μM<br>0.0038 for $[O_2]_{bw}$ < 30 μM
Reference bottom water O_2 for P burial	$[O_2]_{bw}^*$	μM	250
Constant for O_2 dependence on P_{org} burial	α_P	-	0.25

Supplementary References

1 Rao, N. & Torriani, A. Molecular aspects of phosphate transport in Eschericia coli. *Molecular Microbiology* **4**, 1083-1090 (1990).

2 Neidhardt, F. C. *Escherichia coli and Salmonella: Cellular and Molecular Biology*. (ASM Press, 1996).

3 Aguena, M. & Spira, B. Transcriptional processing of the pst operon of Escheria coli. *Current Microbiology* **58**, 264-267 (2009).

4 Adams, M. M., Gómez-García, M. R., Grossman, A. R. & Bhaya, T. Phosphorus deprivation responses and phosphonate utilization in a thermophilic Synechococcus sp. from microbial mats. *J Bacteriol* **190**, 8171-8184 (2008).

5 Lidbury, I. D., Fraser, T., Murphy, A. R., Scanlan, D. J., Bending, G. D., Jones, A. M., Moore, J. D., Goodall, A., Tibbett, M. & Hammond, J. P. The 'known' genetic potential for microbial communities to degrade organic phosphorus is reduced in low-pH soils. *MicrobiologyOpen* **6**, 4 (2017).

6 Canfield, D. E. The early history of atmospheric oxygen: Homage to Robert M. Garrels. *Ann. Rev. Earth Planet. Sci.* **33**, 1-36 (2005).

7 Kump, L. R. & Garrels, R. M. Modeling atmospheric O_2 in the global sedimentary redox cycle. *Am. J. Sci.* **286**, 337-360 (1986).

8 Catling, D. C., Zahnle, K. J. & McKay, C. P. Biogenic methane, hydrogen escape, and the irreversible oxidation of early life. *Science* **293**, 839-843 (2001).

9 Bolton, E. W., Berner, R. A. & Petsch, S. T. The weathering of sedimentary organic matter as a control on atmospheric O2: II. Theoretical modeling. *American Journal of Science* **306**, 575-615 (2006).

10 Denaro, G., Valenti, D., Spagnolo, B., Basilone, G., Mazzola, S., Zgozi, S. W., Aronica, S. & Bonanno, A. Dynamics of two picophytoplankton groups in Mediterranean Sea: Analysis of the deep chlorophyll maximum by a stochastic advection-reaction-diffusion model. *Plos One* **8**, e66765 (2013).

11 Manske, A. K., Glaeser, J., Kuypers, M. M. M. & Overmann, J. Physiology and phylogeny of green sulfur bacteria forming a monospecific phototrophic assemblage at a depth of 100 meters in the Black Sea. *Appl Environ Microb* **71**, 8049-8060 (2005).

12 Vaulot, D., Marie, D., Olson, R. J. & Chisholm, S. W. Growth of Prochlorococcus, a photosynthetic prokaryote, in the equatorial Pacific Ocean. *Science* **268**, 1480-1482 (1995).

13 Jones, C., Nomosatryo, S., Crowe, S. A., Bjerrum, C. J. & Canfield, D. E. Iron oxides, divalent cations, silica, and the early earth phosphorus crisis. *Geology* **43**, 135-138 (2015).
